# Psychometric properties of a clinical reasoning assessment rubric for nursing education

**DOI:** 10.1186/s12912-021-00695-z

**Published:** 2021-09-22

**Authors:** JuHee Lee, Chang Gi Park, Sung Hae Kim, Juyeon Bae

**Affiliations:** 1 Mo-Im Kim Nursing Research Institute, College of Nursing, Yonsei University, Yonsei-ro 50, Seodaemun-gu, Seoul, 03722 South Korea; 2 College of Nursing, University of Illinois at Chicago College of Nursing, 845 S. Damen Ave., MC 802, #612, Chicago, IL 60612-7350 USA; 3 Department of Nursing, Tongmyong University, Sinseon-ro 428, Nam-gu, Busan, 48520 South Korea; 4 Department of Nursing, Yeoju Institute of Technology, Sejong-ro 338, Yeoju-si, Gyeonggi-do 12652 South Korea

**Keywords:** Clinical reasoning, Rubric, Nursing education. Validity, Reliability, Psychometric evaluation

## Abstract

**Background:**

Clinical reasoning is a vital competency for healthcare providers. In 2014, a clinical reasoning assessment rubric (CRAR) composed of analysis, heuristics, inference, information processing, logic, cognition and meta-cognition subdomains was developed for osteopathy students.

**Methods:**

This study was conducted to verify the validity and reliability of the CRAR in nursing education. A total of 202 case vignette assessments completed by 68 students were used for exploratory factor analysis (EFA) and confirmatory factor analysis (CFA). The Cronbach’s α coefficient of the CRAR was calculated.

**Results:**

The content validity indices ranged from 0.57 to 1.0. The EFA resulted in three factors: assessment in nursing, nursing diagnosis and planning, and cognition/meta-cognition in nursing. The CFA supported a 3-factor model. The Cronbach’s α coefficient of the CRAR was 0.94. This study confirmed the content validity, construct validity, and reliability of the CRAR. Therefore, the CRAR is a useful rubric for assessing clinical reasoning in nursing students.

**Conclusions:**

The CRAR is a standardized rubric for assessing clinical reasoning in nurses. This scale will be useful for the development of educational programs for improving clinical reasoning in nursing education.

**Supplementary Information:**

The online version contains supplementary material available at 10.1186/s12912-021-00695-z.

## Background

Nurses use different reasoning processes depending on their knowledge and clinical experiences [1, 2]. According to the Outcome-Present State Test (OPT) model, a theoretical framework for explaining the clinical reasoning process of nurses, accurate awareness of the patient’s overall situation and context is essential in nurses’ decision making and judgment [3]. Nurses use formal and informal thinking strategies such as deliberation and intuition through an individual’s sense of salience, thereby, they use clinical reasoning skills to set priorities in nursing care [4, 5]. Novice nurses use deductive reasoning, which involves using information from the patient and caregiver to draw conclusions. It has been shown that novice nurses do not sufficiently consider salient information, tend to miss important cues, and often focus on the task rather than the patient [2]. In contrast, analytic processes can be used by experts, depending on the context and their knowledge of the situation. They collect data based on previous similar clinical experiences, recognize patient patterns, consider the patient context, and make a complex diagnosis based on these data [1, 2]. Clinical experience with patients and educational level were found to be important factors in relation to nurses’ clinical reasoning [6]. Therefore, nursing students need more effort and education to acquire high-quality clinical reasoning [7].

Under the new learning paradigm that emerged during the industrial revolution, learning occurs through discontinuous semantic relationships and nonlinear thinking processes [8, 9]. Learners connect the perspectives and ideas to which they are exposed lectures and clinical practicums. In this way, academic information is acquired. Clinical reasoning is affected by constructivism [2]. It is defined as a dynamic thinking process to integrate patient data, assess the significance of these data and choose alternative actions [10, 11]. Levett-Jones and colleagues [2] developed the eight cyclic phases of the clinical reasoning model. Nurses used one or more clinical reasoning steps to reach clinical judgements. Clinical reasoning is a problem-solving process that occurs in the clinical context. Due to the uncertainties of patients’ complex health problems, healthcare providers consider a large amount of data when solving patient problems. They need to make decisions using clinical reasoning [11]. Their clinical actions, or judgements, are part of the trajectory of patient outcomes.

The importance of clinical reasoning competency for nurses began to be more emphasized in the US in 2010 [12]. The American Nurses Association (ANA) recommended that clinical reasoning is a required core competency for integrating problem solving in clinical situations. It is a fundamental competency to develop in undergraduate nursing education programs [12]. In addition, having a graded prognostic assessment (GPA) was positively correlated with undergraduate students’ clinical reasoning competency [13]. As a result, educational methods to improve clinical reasoning have been developed. Tyo and McCurry’s integrative review [14] reported that simulation education, active learning strategies such as case studies, and collaborative learning are educational methods that can be used to enhance clinical reasoning*.* Nurses’ clinical reasoning can ensure patient safety by allowing nurses to detect the worsening of symptoms [10]. Generally, clinical reasoning tools have been developed for health care professionals. In medical education, accurate diagnosis is an important aspect of clinical reasoning, while nursing education focuses on clinical reasoning in the nursing process. There is still a lack of sufficient measurement methods for evaluating clinical reasoning competency to support undergraduate nursing student’s education. Liou and colleagues [15] developed a clinical reasoning measurement called the Nurses Clinical Reasoning Scale [NCRC]. The scale emphasizes the logical problem-solving process, which included nurses’ data collection from patients, patients’ recognition of problems, and nursing intervention evaluations. The clinical reasoning tool includes one factor and 15 items that are scored on a Likert scale. The Cronbach’s α of the NCRC was found to be 0.9, indicating its internal consistency. Clinical reasoning is a process used to identify scientific knowledge and evidence to be applied to patients. Nurses continually examine their level of understanding and their cognitive processes; if patient problems occur, nurses reflect and correct mistakes at the reasoning step. Meta-cognition, which is related to understanding and planning, serves to check and regulate nurses’ cognitive states, and it is a key factor in the reasoning process [2]. The NCRC does not include an assessment of cognition and reflection to evaluate nurses’ meta-cognition, which represents a limitation of the NCRC in measuring the core elements of clinical reasoning.

This study is therefore timely; it allows us to deepen our knowledge of the psychometric qualities of the tools. Orrock and colleagues [16] developed a clinical reasoning assessment rubric (CRAR) for osteopathy students based on Simmons’ concept analysis [5] of clinical reasoning. Simmons [5] suggested attributes of clinical reasoning based on the nursing process. However, the construct validity of the CRAR has not been reported. This study was conducted to analyse the validity and reliability of a Korean version of the CRAR (K-CRAR) in nursing education.

## Methods

**Fig. 1:**
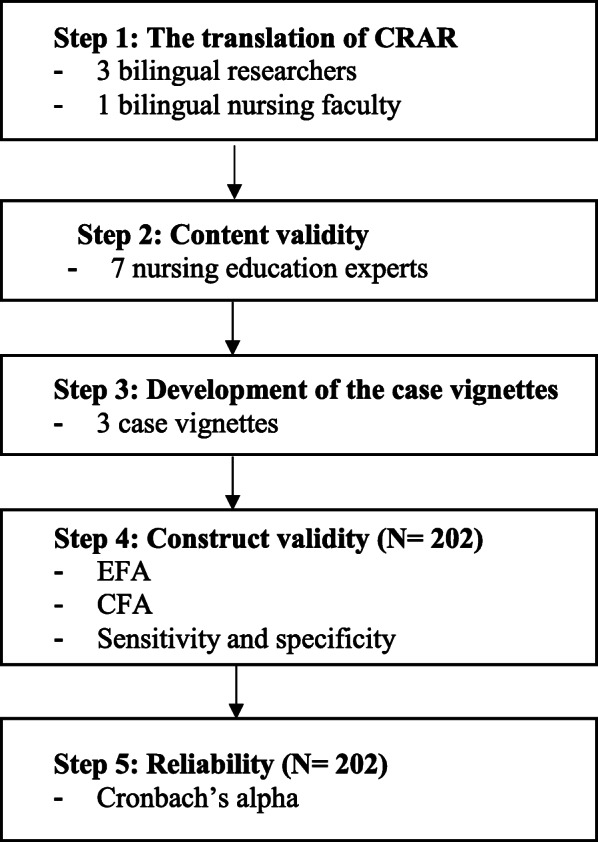
The study process. Note: *CRAR*, Clinical Reasoning Assessment Rubric; *EFA*, exploratory factor analysis; *CFA*, confirmatory factor analysis

This study was conducted to verify the psychometric properties of K-CRAR. In this study, we translated the CRAR into Korean and analysed its validity and reliability. We also developed three types of case vignettes to assess clinical reasoning competency using the CRAR. Our study partially used Kane’s framework to assess the validation process of K-CRAR [17, 18]. Kane’s framework suggested a method of improving the validation rigor. Kane’s framework consisted of domains named as scoring, generalisation, extrapolation, and implications. Extrapolation and implication domains used to examine the validation process. Extrapolation aimed at the measurement domain establishment. At this step, researchers searched the authoritative literature and consulted the experts. The implication domain helped the interpretation of the score using e.g., receiver operating characteristic (ROC). This study procedure as detailed in Fig. 1.

### Instrument

The CRAR is composed of 14 items across 7 subdomains: analysis, heuristics, inference, information processing, logic, cognition, and meta-cognition. Each item of the rubric is scored from 1 to 5 points, with a total score ranging from 14 to 70. A higher score indicates a higher level of clinical reasoning. The internal consistency test of the rubric reported a Cronbach’s α = 0.944 [16].

### Translation process

The original CRAR was translated into Korean by the researchers after permission was obtained from the author [19]. The translation process was performed according to World Health Organization guidelines [20]. First, three bilingual researchers, including nursing professor, translated the text into Korean. Each researcher translated the CRAR independently. The translated K-CRAR was then revised by the researchers through a review of the items to consider the accuracy of the translation. During this process, the K-CRAR was translated into English for comparison to the original by bilingual nursing faculty who were experienced in psychometric validation. The preliminary K-CRAR draft was then completed.

### Content validity

The content validity was examined by 7 nursing education experts who reviewed each rubric item via email in a one-round panel. The experts individually analysed the suitability of the content in terms of the language equivalence between the original and translated rubric. The experts were selected based on a list of faculties suggested by the Korean Accreditation Board of Nursing Education [21]. The experts verified the validity of the items by indicating the appropriateness of each item on a Likert scale from 1 (“not at all”) to 4 (“very appropriate”). The experts commented about items that needs revision. The content validity index (CVI) was calculated, and items with 80% agreement were selected [22].

### Development of the case vignettes

Case vignettes were developed based on nursing textbooks [23, 24]. We developed different types of case vignettes with different difficulty levels from easy to difficult. A pilot study was performed to estimate the 7 subdomains of the K-CRAR to ensure inter-rater agreement for the three cases. The researchers discussed points of disagreement until reaching a consensus. An example of a case vignette is shown in Additional file 1.

### Construct validity

The construct validity was verified using exploratory factor analysis (EFA) and confirmatory factor analysis (CFA). The sensitivity and specificity were identified using a ROC curve to calculate the cut-off point.

### Reliability

The internal consistency was examined using Cronbach’s α.

### Participants and procedure

The required sample size was calculated using a method based on the root mean square error of an approximation method, which involved calculating the number of samples for the power in the factor analysis. For 70 degrees of freedom, a power of 0.8, and an α of 0.05, the required sample size was 200. Each participant completed three case vignettes, resulting in three samples per participant. Thus, at least 67 participants were needed, and a total sample size of 70 was used in case of dropouts. The inclusion criteria were 4th-year nursing students who had completed their clinical practicum courses and were able to understand the vignette cases and the purpose of the study. Participants were recruited from three universities in Korea from June to December 2018. A total of 68 4th-year nursing students participated in this study. One participant did not complete the assessment for two case vignettes. Therefore, a total of 202 case vignette assessments were included in the final analysis.

### Ethical considerations

Ethical approval was provided by the Yonsei University College of Nursing Ethics Committee (2016–0028). Participants were recruited from three universities in Korea from June to December 2018. We obtained written informed consent from all participants prior to study enrolment.

### Statistical analysis

The data were analysed using SPSS 23.0 and AMOS version 21.0. Descriptive statistics were used to analyse the participants’ demographic characteristics. In this study, we performed validity and reliability tests. First, an item analysis was conducted. Second, the construct validity was identified using EFA and CFA. A principal component analysis and the varimax rotation method were used. The criteria of factor loadings were greater than 0.5 and a *p* value less than 0.05 [25]. We used structural equation modelling for the CFA. The model fit criteria were as follows: chi-squared/degrees of freedom (χ^2^/df) < 3.0, goodness-of-fit index (GFI) > .90, comparative fit index (CFI) > .90, normed fit index (NFI) > .90, Tucker-Lewis index (TLI) > .90, and standardized root mean residual (SRMR) < .08 [26, 27]. Third, the sensitivity and specificity were analysed using the area under the curve (AUC) on the ROC curve with cut-off point calculation. Hosmer and Lemeshow [28] reported a general rule for the interpretation of the AUC using the ROC curve as follows: AUC = 0.5 indicates no discrimination, 0.7 ≤ AUC < 0.8 represents acceptable discrimination, 0.8 ≤ AUC < 0.9 represents excellent discrimination, and AUC ≥ 0.9 suggests outstanding discrimination. Lastly, the internal consistency and reliability were assessed using Cronbach’s α. This study consulted the statistician to corroborate the findings.

## Results

### Content validity

The CVI of the K-CRAR was 0.92, ranging from 0.57 to 1.00 for each question. The experts commented on the meaning of “differential diagnosis” in the original tool. The preliminary K-CRAR did not include a differential diagnosis in the nursing process. However, the experts suggested that diagnostic approaches are an important skill for nurses. When patients have diverse and complex diseases, nurses should use multidimensional thinking strategies to analyse and distinguish patient information that can lead to decision making [12, 29]. Therefore, diagnostic identification was added to the rubric.

The experts suggested that the response option for item #10 should be written as ‘nursing intervention’ rather than ‘nursing diagnosis’. Based on the experts’ comments, we rephrased item #10 to include the term ‘nursing intervention’.

### General characteristics and K-CRAR scores

The general characteristics of the participants, including their K-CRAR scores, are shown in Table 1. The mean age of the nursing students was 22.53 ± 1.4 years. Of the 68 participants, 94.1% were female. Aptitude and interest in nursing were the most frequent motivation factors for entering college (*n* = 29, 42.6%). Most participants were satisfied with their major (*n* = 44, 64.7%) and clinical practicum (*n* = 40, 58.8%). There was no significant difference in the clinical reasoning scores according to the participants’ general characteristics. The scores ranged from 23 to 70, and the mean score was 50.47 ± 8.93.

**Table 1:** Demographic characteristics of the study sample

	Participants (*n* = 68) N (%) or Mean (SD)	K-CRAR Score (*n* = 202) N (%) Mean (SD)		*t* or F (*p*)
Age, mean	22.53 (1.4)		3.61 (0.6)	
Age (years)				
≤ 21	13 (19.1)	39 (19.3)	3.73 (0.6)	1.661
22–23	42 (61.8)	124 (61.4)	3.61 (0.7)	(.193)
≥ 24	13 (19.1)	39 (19.3)	3.47 (0.5)	
Gender				
Male	4 (5.9)	12 (16.8)	3.52 (0.7)	−.487
Female	64 (94.1)	190 (83.2)	3.61 (0.6)	(.627)
Admission Motivation				
School grade	5 (7.4)	15 (7.4)	3.51 (0.6)	2.247
Recommendation & Advice	14 (20.6)	42 (20.8)	3.74 (0.7)	(.065)
Employment	11 (16.2)	31 (15.3)	3.33 (0.7)	
Aptitude & interest	29 (42.6)	87 (43.1)	3.66 (0.6)	
Profession	9 (13.2)	27 (13.4)	3.58 (0.5)	
Major Satisfaction				
Very satisfied	14 (20.6)	42 (20.8)	3.60 (0.6)	0.012
Satisfied	44 (64.7)	132 (65.3)	3.61 (0.6)	(.988)
Moderate	10 (14.7)	28 (13.9)	3.59 (0.7)	
Clinical Practicum Satisfaction				
Very satisfied	6 (8.8)	18 (8.9)	3.85 (0.5)	1.742
Satisfied	40 (58.8)	120 (59.4)	3.56 (0.6)	(.160)
Moderate	20 (29.4)	58 (28.7)	3.59 (0.7)	
Dissatisfied	2 (2.9)	6 (3.0)	3.95 (0.6)	
Academic Achievement				
High - Middle	25 (36.8)	75 (37.1)	3.74 (0.7)	2.843
Middle	24 (35.3)	70 (34.7)	3.55 (0.6)	(.061)
Middle - Low	19 (27.9)	57 (28.2)	3.50 (0.6)	

*Abbreviations*: *K-CRAR* Korean version of Clinical Reasoning Assessment Rubric, *SD* standard deviation

### Validity – exploratory factor analysis

In Table 2, the EFA results are reported. The Kaiser-Meyer-Olkin coefficient was 0.84, and Bartlett’s test of sphericity was significant at *p* < 0.001, which was appropriate for factor analysis. All the factor loadings were significant (*p* < 0.05) and above 0.5. Factors with eigenvalues greater than 1.0 were extracted. Overall, three factors explained 75.19% of the total variance in the 14 items. The eigenvalues of the three factors were 7.88, 1.43, and 1.22, and these factors explained 56.26, 10.19, and 8.74% of the variance, respectively.

**Table 2:** Exploratory factor analysis of K-CRAR according to component analysis (*N* = 202)

K-CRAR item	Mean		SD	Cronbach’s Alpha if Item Deleted	Corrected Item-Total Correlation	Factor Loading			Eigenvalue	Variance (%)	Cumulative variance (%)
						Component 1	Component 2	Component 3			
Item 9	3.64	±	0.84	0.94	0.70	0.88	0.19	0.17			
Item 8	3.68	±	0.85	0.93	0.77	0.86	0.28	0.22			
Item 10	3.59	±	0.85	0.94	0.69	0.85	0.18	0.20			
Item 7	3.45	±	0.87	0.93	0.79	0.65	0.32	0.47			
Item 6	3.58	±	0.84	0.93	0.73	0.64	0.31	0.37			
Item 5	3.58	±	0.85	0.93	0.76	0.56	0.55	0.25	7.88	56.26	
Item 2	3.23	±	0.82	0.94	0.66	0.18	0.86	0.19			
Item 3	3.61	±	0.92	0.93	0.77	0.29	0.82	0.29			
Item 1	3.38	±	0.87	0.94	0.69	0.20	0.81	0.28			
Item 4	3.51	±	0.95	0.93	0.75	0.32	0.79	0.27	1.43	10.19	66.46
Item 14	4.10	±	0.73	0.94	0.61	0.11	0.24	0.85			
Item 13	4.02	±	0.82	0.94	0.69	0.26	0.25	0.81			
Item 11	3.65	±	0.86	0.94	0.64	0.32	0.23	0.69			
Item 12	3.44	±	0.86	0.94	0.57	0.25	0.21	0.66	1.22	8.74	75.19

*Note*: *K-CRAR* Korean version of Clinical Reasoning Assessment Rubric, *SD* standard deviation

The scree plot showed a three-point threshold, and the plot dropped sharply after the first point. We renamed the factors according to their properties. The first factor was named “diagnosis and planning in nursing.” It consisted of the inference, information processing, and logic subdomains of the original CRAR (items 5 to 10). The second factor was named “assessment in nursing,” which was composed of the analysis and heuristics subdomains of the original CRAR (items 1 to 4). Lastly, the third factor was named “cognition and meta-cognition in nursing,” which included the subdomain of the original CRAR (items 11 to 14).

### Validity – confirmatory factor analysis

**Fig. 2:**
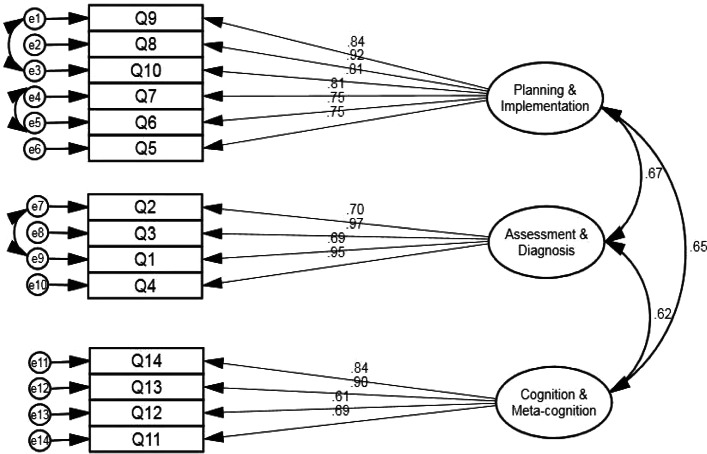
Confirmatory factor analysis results of K-CRAR. Note: *K-CRAR*, Korean version of Clinical Reasoning Assessment Rubric

We analysed the model fit. The fit indices of the three-factor model were not adequate (χ^2^/df = 5.619, GFI = .774, CFI = .858, NFI = .834, TLI = .826, and SRMR = .091). We modified the model using the modification index, which showed covariance within the same factor as follows: errors 1 and 3, errors 4 and 5, and errors 7 and 9. The revised model fit indices, excluding the GFI, were adequate (χ^2^/df = 2.984, GFI = .859, CFI = .942, NFI = .915, TLI = .925, and SRMR = .079) (Fig. 2).

### Sensitivity and specificity

**Fig. 3:**
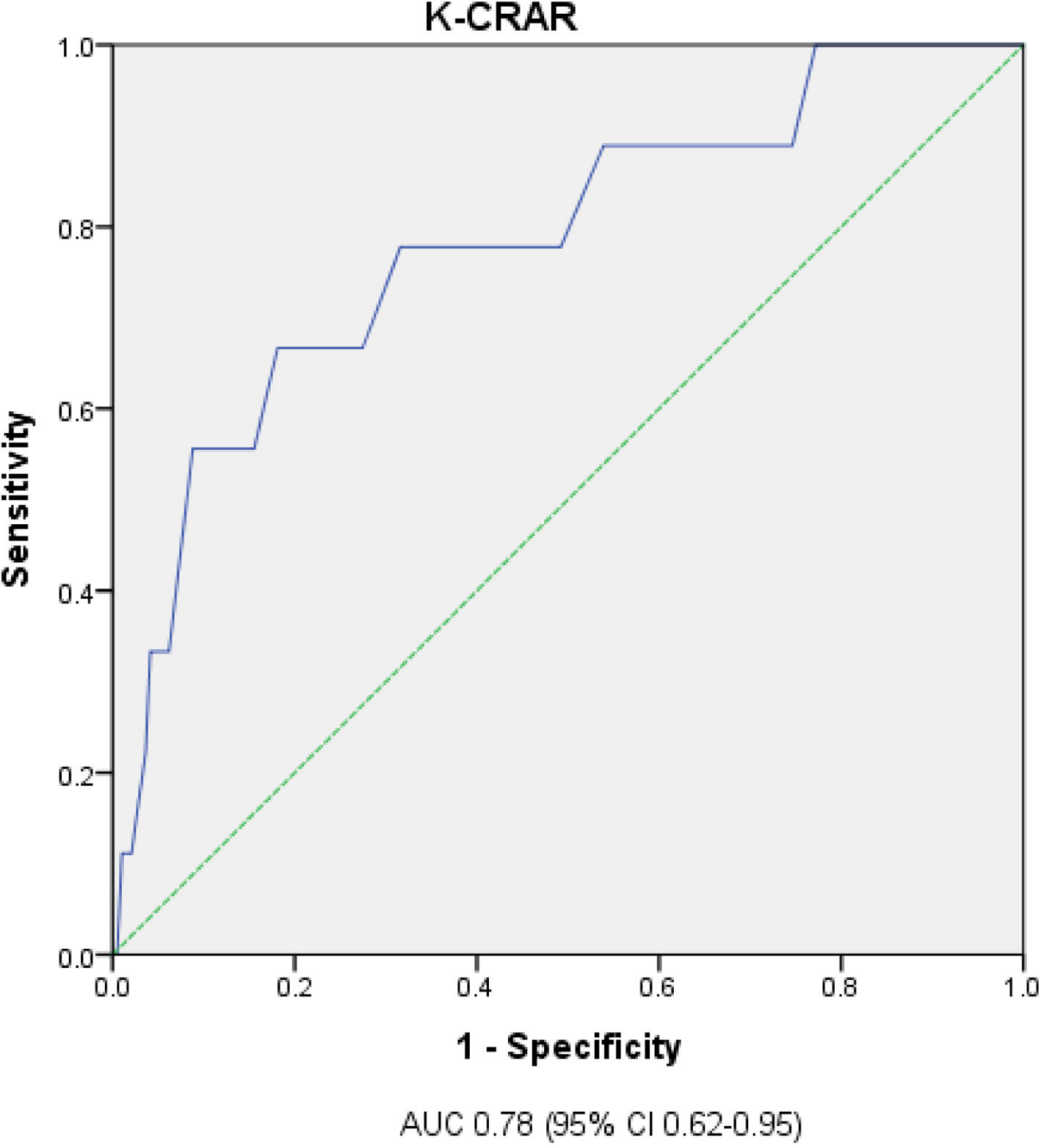
ROC curves and AUC for GPA grade score according to K-CRAR. Note: *AUC*, Area Under Curve; *K-CRAR*, Korean version of Clinical Reasoning Assessment Rubric; *GPA*, Graded Prognostic Assessment; *ROC*, Receiver Operating Characteristic

The sensitivity and specificity of the K-CRAR were tested using cumulative overall GPA scores. The GPA consisted of required and elective subjects, in addition to liberal arts points. We applied a cut-off point of a GPA grade of A^+^. The ROC curve and AUC are shown in Fig. 3. The AUC was 0.78 (95% CI 0.62–0.95, *p* = 0.004). This result showed that there was acceptable discrimination (> 0.05) and that the K-CRAR was suitable for screening [29]. To assess nursing students with high clinical reasoning, the cut-off level was set at 56. The sensitivity was 66.7%, and the specificity was 27.5%.

### Reliability

For the 14 items of the K-CRAR, the Cronbach’s α coefficient was 0.94, indicating excellent internal consistency (*N* = 68, 202 cases). The three subscales presented adequate internal consistency: 0.93 for diagnosis and planning, 0.92 for assessment, and 0.84 for cognition and meta-cognition.

## Discussion

The importance of this study was that it suggested the standardized rubric to estimate clinical reasoning in nursing education. By providing the verified rubric, the study results will contribute to the development of educational programs for improving nurses’ clinical reasoning. Originally, the CRAR was developed to measure the clinical reasoning of osteopathy students; however, the authors did not report the psychometric validation of the rubric. This study was conducted to evaluate the content validity, construct validity, and reliability of the K-CRAR to verify whether it can be used to measure clinical reasoning in undergraduate nursing students. This study provided a foundation for applying the clinical reasoning rubric in nursing.

All of the items of the K-CRAR except for one were confirmed suitable for measuring the clinical reasoning of nursing students [30]. The lowest CVI was for item # 10, which asked whether the learner could make a diagnosis and present a rationale for nursing intervention strategies. The content validity of the rubric indicates that it appropriately reflects the content or theme of measuring clinical reasoning. The accurate interpretation of terminology is important for verifying the content validity. The interpretation of terminology affects the rigor and completeness of an instrument [17]. In the case of item 10, the question and scoring criteria were phrased differently between the original and the translation; therefore, the item description was revised to improve the interpretation. In addition, nurses’ evidence-based diagnostic competency was found to lead to positive outcomes for patients [11, 31]. Therefore, the items evaluating accurate diagnosis, skills and strategies were maintained in the K-CRAR.

In case of measurements are translated into other languages, CFA is appropriate for construct validity [32]. Factor analysis was not performed for the CRAR during the development stage. Therefore, EFA and CFA were conducted to verify the construct validity of the rubric. Our study performed an EFA to identify the number of factors [33]. The original rubric consisted of seven subdomains with 1 to 3 items per factor. According to the EFA, the Korean version consisted of three subdomains. We renamed the factors according to aspects of the nursing process. The diagnosis and planning factor had the highest explanatory power, at 56.26%. The nursing process is a systematic approach to nurses’ problem solving [34]. This approach leads to problem identification in the overall context. Nurses’ systematic thinking guides complex causality and accurate judgement [3]. This factor reflected the most effective, systematic aspect of clinical reasoning. The three factors of K-CRAR explained the 75.19% result. This is a similar result to previous psychometric testing of NCRC, a clinical reasoning scale. The NCRC’s explanatory power was 50.66%, and the Korean version of the NCRC was 61.63% [5, 35]. The K-CRAR satisfied Streiner’s [36] criteria. Streiner proposed an explanatory power of at least 50 [36]. Overall, these findings indicate that the K-CRAR is an appropriate rubric for assessing clinical reasoning competency.

In this study, the CFA did not indicate a good fit of the model. We analysed the items connected to similar items the same attribute considering the covariance. The revised model fit indices, excluding the GFI, were adequate. The rubric could measure the clinical reasoning of nurses using all items and subscales. A modified three-factor measure was adopted for the final model. This model provided a possible explanation for why the theoretical model was not supported by the CFA. Further research is necessary to conduct repeated studies using CFA and EFA to provide an improved model.

The internal consistency of the test showed that the Cronbach’s α for the overall measure was .94. The Cronbach’s α coefficients of the subdomains ranged from 0.84 to 0.93. The Cronbach’s α of the original CRAR was .94. Compared to other clinical reasoning tools, the NCRC Cronbach’s α was .94, and the Korean version of the NCRC was .93 [3, 16]. The rubric had high reliability among the participants of this study [37].

The AUC of the K-CRAR was 0.78. This rubric had moderate accuracy according to Greiner et al.’s [38] standard. Similarly, Suebnukarn & Haddawy [39] developed a problem-based learning model for medical students that included clinical reasoning processes such as problem identification, problem analysis, hypothesis reporting and that had an AUC of 0.878, which was consistent with this study. The cut-off score is a significant indicator for evaluating scale accuracy in screening measurements. A cut-off value might be calculated to increase sensitivity [40]. It is appropriate to select cut-off values for both high sensitivity and specificity. The sensitivity of the K-CRAR was 66.7%, and the specificity was 27.5%, with a cut-off value of 56. This result might have been due to the difficulty of the vignette cases. A factor potentially affecting the difficulty of the cases was the time elapsed since the participants had completed their clinical practicums. A previous study reported that students’ acquired knowledge significantly decreased 8 weeks later after completing clinical practicums [41]. The study results may have been affected by recall bias. Despite the results, this study used diverse methods to examine the validity and reliability of the rubric. The K-CRAR met the optimal cut-off point. This rubric is useful for evaluating the clinical reasoning of nursing students.

### Limitations

The limitations of this study are as follows. First, the validity and generalizability of the results are limited because the sample comprised only undergraduate nursing students. Thus, a verification of the results with diverse samples (i.e., nurses or nurse practitioners) is suggested in the future*.* Second, the selected cut-off point was optimal. This was a multisite study. There were differences in the GPA level calculations by school. Thus, when this rubric is used, it is possible to change the cut-off point, and it is necessary to carefully interpret the results.

## Conclusions

Clinical reasoning is a core competency for nurses. It is needed to emphasize fostering clinical reasoning in undergraduate nursing education. We verified a rubric for measuring the clinical reasoning of nursing students. The use of a standardized rubric enables the accurate and objective assessment of clinical reasoning. A standardized tool could be used to effectively measure the extent to which a researcher’s intervention has made a difference.

It is important to develop educational programs to promote nurses’ clinical reasoning so they can support people with health problems. This rubric provides an objective indicator of clinical reasoning development in education.

## Supplementary Information

**Additional file 1.** Case vignette.

## Data Availability

The authors confirm that all the relevant data are included in the article and its supplementary information files.

## Abbreviations

CRAR: Clinical reasoning assessment rubric
EFA: Exploratory factor analysis
CFA: Confirmatory factor analysis
ANA: The American Nurses Association
NCRC: Nurses Clinical Reasoning Scale
CVI: Content validity index
ROC: Receiver operating characteristic
GFI: Goodness-of-fit index
CFI: Comparative fit index
NFI: Normed fit index
TLI: Tucker-Lewis index
SRMR: Standardized root mean residual
AUC: Area under the curve
GPA: Graded prognostic assessment
