# Supplementary material for: Psychometric properties of a clinical reasoning assessment rubric for nursing education
Source: BMC Nurs. 2021 Sep 22;20:177. doi: 10.1186/s12912-021-00695-z (PMC8456525; doi:10.1186/s12912-021-00695-z)
Supplement: Supplementary file 1 — Additional file 1. Case vignette. [file 12912_2021_695_MOESM1_ESM.docx]

**Case vignette: Acute MI version**

*Student instruction*

You are a nurse working at a senior general hospital. Read the following scenario and answer the questions below.

**1. Patient history**

**1) Chief complaint**

“I feel stuffy in my chest.”

**2) Present illness**

Kim is a 41-year-old-male. He visited the emergency department with complaints of chest discomfort, heartburn, shortness of breath and severe chest pain in the lower part of the sternum. His VAS pain score is 7 points. Kim has smoked 1/2 pack (10 cigarettes) per day for 10 years, and recently, he has been smoking more often than usual. He has been chronically coughing while smoking and occasionally has chest pain when coughing, but this is the first time he has felt as much pain as today. Kim has been under a great deal of stress, and he was very anxious about sudden chest pain. There was no fever, chills, dysphoria, nausea or vomiting.

**3) Past illness**

▪ diagnosed with panic disorder while serving in the military (22 years old)

▪ diagnosed with chronic gastritis (35 years old)

▪ No other diseases have been specifically diagnosed, including cancer

▪ Surgical history: None

▪ Allergies: No allergies such as food, drugs, latex, etc.

**4) Medication history**

▪ He was 22 years old when he started taking medication for panic disorder, which he took for 5 years.

▪ He takes chronic gastritis medication sometimes.

**5) Family history**

▪ His mother (53 years old) was diagnosed with hypertension

▪ His father died of stomach cancer at the age of 52.

**6) Social-economic status**

▪ Marital status: married

▪ Alcohol: drinks three to four drinks per week

▪ Smoking: 1/2 pack (10 cigarettes) per day for 10 years

***Student Worksheet Ⅰ***

| **Q1. What else should you ask Mr. Kim to add to his health history?**  **Q2. What type of assessment are you going to implement for Mr. Kim?**  **Q3. What is the currently inferred health problem for Mr. Kim?** |
| --- |

**2. Health Assessments**

**1) Review of System**

▪ Cardiac: No palpitations, syncope, dizziness, lightheadedness, severe chest pain (+), chest pain at resting (+)

▪ Pulmonary: Shortness of breath (+), chronic cough (+), hemoptysis (-), wheezing (-),

▪ Endocrine, Musculoskeletal, Neurologic: No specific change.

▪ Gastrointestinal tract: Nausea (-), vomiting (-), diarrhea (+/-), anorexia (-), epigastric pain (+)

**2) Physical examinations**

▪ Height: 175 cm

▪ Weight: 71 kg

▪ V/S

- BP: 168/90 mmHg, PR: 110 rate/min, RR: 28 rate/min, BT: 36.5℃, SpO_2_ 95%

▪ Chest

- Heart sounds regular (no murmur)
- severe chest pain in the lower part of the sternum with cramping

▪ CXR: No abnormal finding

▪ No other specific finding

***Student Worksheet Ⅱ***

| **Q1. What is the currently inferred health problem for Mr. Kim?**  **Q2. What additional assessment or tests are needed for Mr. Kim?** |
| --- |

**3. Examination Findings**

**1) 12 lead EKG**

▪ An electrocardiogram performed in the emergency room shows ST elevation (3∼5 mm) in precordial leads V3-6

**2) Cardiac enzymes**

| Labs | Most Recent | Normal Range |
| --- | --- | --- |
| CPK | 542 IU/L | < 190 IU/L (M) < 170 IU/L (F) |
| LDH | 475 IU/L | 140~280 IU/L |
| CK-MB | 36.44 IU/L | 0~4.87 IU/L (M) 0~3.61 IU/L (F) |
| Troponin-T | 0.489 ng/mL | 0~0.1 ng/mL |

***Student Worksheet Ⅲ***

| **Q1. What is the final health problem for Mr. Kim?**  **Q2. What are the medication treatments needed for Mr. Kim?**  **Q3. What are the priorities for nursing practice?**  **Q4. What additional assessment, tests, and treatments should be performed?** |
| --- |
